# Evaluation of additional causes of hip pain in patients with femoroacetabular impingement syndrome

**DOI:** 10.3389/fsurg.2022.697488

**Published:** 2022-08-10

**Authors:** Anirudh K. Gowd, Edward C. Beck, Amy P. Trammell, Carl Edge, Allston J. Stubbs

**Affiliations:** Department of Orthopedic Surgery, Wake Forest University Baptist Medical Center, Winston-Salem, NC, United States

**Keywords:** FAI (Femoroacetabular impingemet), sacroiliac pain, lumbosacral pain, osteitis pubis, pseudoradicular pain

## Abstract

Femoroacetabular impingement syndrome (FAIS) is an increasingly prevalent pathology in young and active patients, that has contributing factors from both abnormal hip morphology as well as abnormal hip motion. Disease progression can be detrimental to patient quality of life in the short term, from limitations on sport and activity, as well as the long term through early onset of hip arthritis. However, several concurrent or contributing pathologies may exist that exacerbate hip pain and are not addressed by arthroscopic intervention of cam and pincer morphologies. Lumbopelvic stiffness, for instance, places increased stress on the hip to achieve necessary flexion. Pathology at the pubic symphysis and sacroiliac joint may exist concurrently to FAIS through aberrant muscle forces. Additionally, both femoral and acetabular retro- or anteversion may contribute to impingement not associated with traditional cam/pincer lesions. Finally, microinstability of the hip from either osseous or capsuloligamentous pathology is increasingly being recognized as a source of hip pain. The present review investigates the pathophysiology and evaluation of alternate causes of hip pain in FAIS that must be evaluated to optimize patient outcomes.

## Introduction

Hip pain is prevalent in the athletic population, comprising approximately 5%–6% of adult sports injuries and 10%–24% of pediatric sports injuries (1, 2), though the exact source of pain has many possible sources. Femoroacetabular impingement syndrome (FAIS), generally defined as the abnormal contact between femur and acetabulum, has recently garnered increased attention as being responsible for the majority of hip pathologies in the pre-arthritic population (3). It has also become evident that hip morphology plays a significant role in the development of hip osteoarthritis, even if asymptomatic (4, 5). As such, utilization of hip arthroscopy is increasingly utilized for management of hip conditions, most often FAIS, and indications continue to rapidly evolve (6–8). Despite the focus on FAIS, alternative and concurrent pathologies must also be considered as concomitant causes of hip pain. The diagnosis of pre-arthritic hip pathology is complex, and failure to address a contributing cause may result in inferior outcomes and additional surgical procedures. The purpose of this review is to highlight other potential concomitant sources of pain in patients with FAIS.

## Imbalance in spinopelvic alignment and sacroiliac pathology

**Figure 1:**
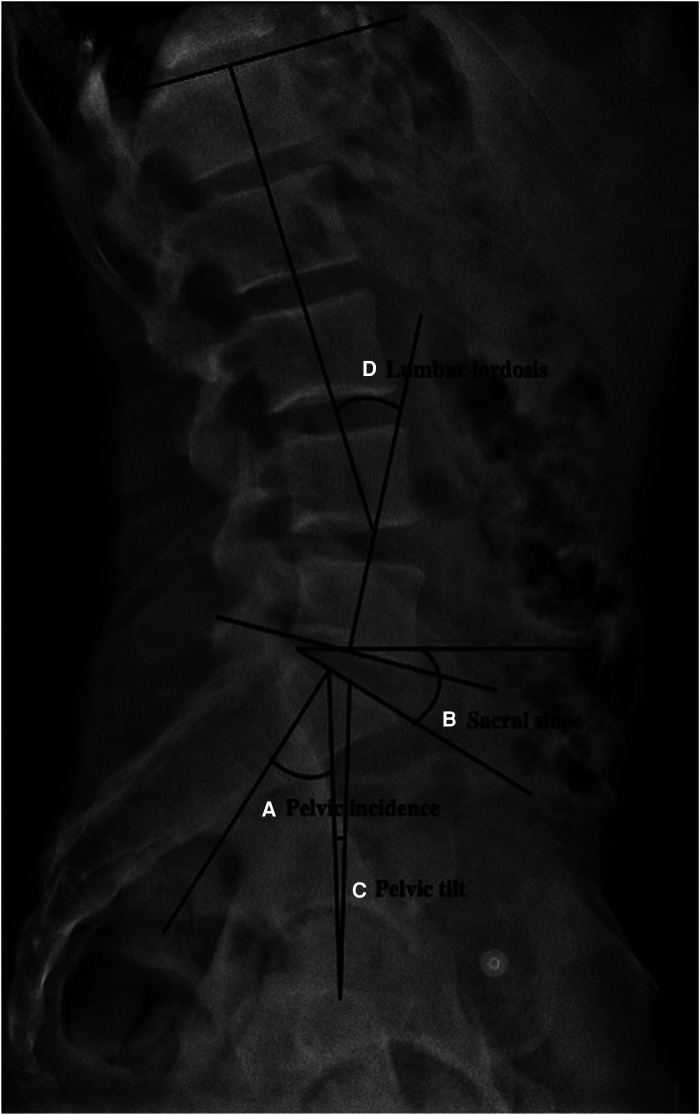
Radiographic measurements of (**A**) pelvic incidence, (**B**) sacral slope, (**C**) pelvic tilt, and (**D**) lumbar lordosis from lateral lumbar view radiograph.

The role of the pelvis in sagittal balance has been an area of increased recent investigation. Spinopelvic parameters including pelvic tilt, sacral slope, and pelvic incidence influence how patients can distribute weight across their axial and appendicular skeletons (Figure 1). Of which, pelvic incidence is the only parameter that is independent of position (9). Importantly, the greater the pelvic incidence, a greater lumbar lordosis (particularly in the proximal segment) is required to maintain an upright posture (10). Patients with low pelvic incidence are theorized to compensate with increased forward tilt of the pelvis. This motion likely results in over-coverage of the anterior acetabulum, and thereby, places this pelvis prone to impingement in the hip joint (11). Gebhart et al. postulated the delicate balance of pelvic incidence wherein higher incidence places more mechanical force on the lumbar spine, while lower incidence places greater force through the hip joint with associated cam and pincer morphologies (9). These authors analyzed cadaveric specimen to support this hypothesis and found both cam and pincer-morphologies were associated with decreased pelvic incidence (9). Weinberg et al. corroborated these findings clinically, suggesting that decreased pelvic incidence is correlated with mixed-type FAI (12). Fader et al. explored this relationship further and observed that symptomatic FAIS patients had greater sacral slope when sitting, and a compensatory increase in pelvic tilt (13). This illustrates the concept that lumbopelvic stiffness may result in compensatory greater hip flexion, and thereby, a greater propensity for cam lesions to engage the acetabulum (13). Recent evidence would thereby suggest that patients with lumbar pathology or surgery, particularly fusion, have inferior clinical outcomes following hip arthroscopy (14–16).

In addition to identifying potential spinopelvic sagittal imbalances, a thorough examination of the sacroiliac joint should be performed as it has potential to elicit generalized hip pain. The sacroiliac joint plays a pivotal role in spinopelvic biomechanics by absorbing torsion and transferring load during movement. Previous studies have demonstrated that patients with altered hip range of motion have evidence of sacroiliac dysfunction (17). Altered range of motion may be caused by muscular imbalances, particularly hip rotators and extensors, but may also be caused by bony abnormalities leading to impingement. Indeed, a recent study by Krishnamoorthy and colleagues demonstrated that patients who underwent hip arthroscopy for treatment of FAIS and demonstrated SI joint abnormalities incidentally found on preoperative radiographs had lower outcomes when compared to patients who did not have SI joint abnormalities (18). Other studies have indicated that 17%–25% of patients have concomitant hip and SI joint pathology (18, 19). The growing evidence of concomitant lumbosacral pathology in patients with FAIS and other hip abnormalities may indicate a causal relationship between both that should be further evaluated in future studies.

Work-up for evaluation of spinopelvic derangements should include standing lumbar AP and lateral radiographs in order to evaluate spinopelvic derangements. Clinical findings such as positive FABER (flexion abduction, and external rotation) test, sacroiliac joint shear test, or Gaenslen's test may point to the SI joint as a cause for hip pain (20). While no single provocation test can accurately identify pain related to SI dysfunction, positive response to 3 or more maneuvers has a sensitivity of 77%–87% (21). Additional imaging studies including MRI may be ordered if the SI joint is suspected to be a source of hip pain, as it is the most sensitive imaging technique for detecting sacroiliitis (22).

## Athletic pubalgia and osteitis pubis

Athletic pubalgia is a pathology often affecting those participating in sports with repetitive pivoting and cutting including soccer, and hockey (23). The rectus abdominis and hip adductor tendons (pectinius, gracilis, adductor longus/brevis and magnus) attach to the pubic ramus and provide pelvic stability. Athletic pubalgia is defined as injury to these musculotendinous structures near their bony insertion, resulting in pain and instability. It frequently coexists in patients with FAIS, reportedly in as high as 43.48% of patients with FAIS (24–27). Patients with decreased hip motion from FAIS are believed to compensate in motion through the pubic symphysis (28). The compensatory increased motion through the pubic symphysis causes increased stress and strain that can result in pathology to the symphysis (29, 30).

**Figure 2:**
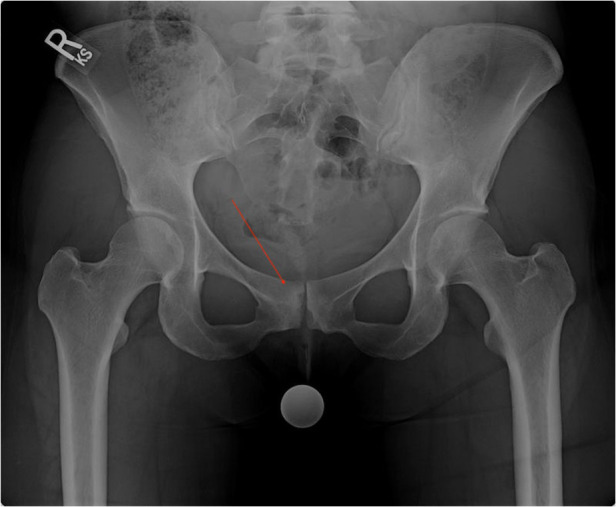
Anteroposterior radiographic image of a pelvis demonstrating bone edema (red arrow) and sclerotic changes in the pubic symphysis consistent with osteitis pubis.

Chronic compensatory movement of the pubic symphysis can lead to osteitis pubis, defined by inflammatory changes in the joint (Figure 2). Previous studies have demonstrated that FAIS patients with pubic symphysis abnormalities on MRI had inferior post-operative functional outcomes after arthroscopic treatment of FAIS impingement (18). While the prevalence of osteitis pubis was low (2.3%), the presence of this pathology has the potency to limit functional gain from surgery if not addressed (18). These findings are supported by Birmingham and colleagues who found that repetitive loading of the symphysis secondary to cam morphology and impingement causes increased motion at the pubic symphysis (31).

Examination findings in patients with athletic pubalgia or osteitis pubis include pain with palpation of the pubis, inferior rectus abdominis, or adductor muscles, as well as exacerbated with resisted sit-ups at the inferolateral edge of the rectus abdominis (23). Tears and injury to other nearby musculotendinous structures including rectus femoris and iliopsoas tendinosis are associated with anterior hip pain as well (32). Test's including FABER test and tenderness over Scarpa's triangle may indicate either rectus femoris or iliopsoas tendonitis (32). A positive spring test may help differentiate osteitis pubis to that of athletic pubalgia. This test may be performed through palpation of the pubic rami, and results in pain in the symphysis (23). However, advanced imaging modalities, including MRI, may be necessary to confirm the presence of both and assist in directing further management. Previous studies have indicated that MRI has high specificity in identifying tendon tears associated with sports hernia, as well as osteitis pubis (33).

## Impact of femoral and acetabular version

FAIS is typically recognized as impingement caused by acetabular over-coverage (pincer lesion), bony morphological changes at the femoral head-neck junction (cam lesion), or a combination of both. However, deviations in acetabular and femoral version may also cause impingement that may often go overlooked in traditional 2-dimensional radiographic workup. While the definition of acetabular retroversion remains debated, it is associated with crossover sign, ischial spine sign, and posterior wall sign (34, 35). Previous studies have demonstrated that patients with acetabular retroversion have higher rates of sub-spine impingement as well as larger femoral head coverage, when compared to patients with normal acetabular version (34). Historically, reverse periacetabular osteotomy has been used as the gold standard for treating acetabular retroversion, however, recent studies have demonstrated that patients with labral tears in the presence of acetabular retroversion can be treated arthroscopically without the need of acetabular osteotomies (36).

Femoral version may also play a role in FAIS not associated with traditional cam or pincer lesions. Recently, Lerch and colleagues determined that hips with decreased femoral version have decreased range of motion, specifically hip flexion and internal rotation in 90° of flexion (37). Additionally, the authors observed decreased femoral version was associated with both intra- and extraarticular impingement. Previous studies have evaluated whether femoral version has an impact on outcomes in patients who underwent arthroscopic treatment for FAI syndrome. Fabricant et al. analyzed outcomes among 243 patients and when stratified by femoral version, the authors observed that patients with femoral retroversion had statistically smaller magnitudes of postoperative improvements when compared to patients with normal femoral version (38). However, the literature is limited on whether solely addressing femoral or acetabular version in the absence of labral tears improves patient function or improves hip biomechanics.

Physical evaluation of abnormal femoral torsion includes assessing internal and external range of hip motion. Previous studies have found that patients with greater external rotation are associated with retroversion while greater internal rotation are associated with femoral neck anteversion (39). However, these measurements can be subjective and not diagnostic, particularly in adults (40). Provocative maneuvers during the clinical exam can assist in further evaluation of femoral torsion including the trochanteric prominence angle test (TPAT) also know as the Craig's test (41). Diagnosis and measurement of femoral neck torsion is performed typically using CT images by measuring the angle formed between a line down the middle of the femoral neck and a line parallel to the posterior aspect of the femoral condyles (42). The normal version of the femur is anteversion, with a normal range of approximately 10–20 degrees (17, 42).

**Figure 3:**
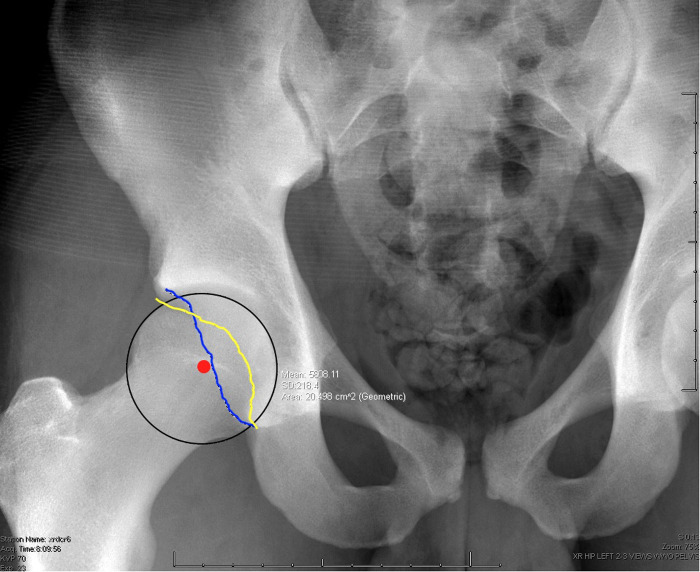
Anteroposterior radiograph of the hip demonstrating the cross oversign associated with acetabular retroversion. In the image the yellow line represents the anterior rim of the acetabulum, blue line reprents the posterior rim, and the red dot is the middle of the femoral head.

Clinical evaluation of acetabular retroversion can be challenging in the absence of concomitant conditions (i.e. SCFE, dysplasia). Trochanteric pain with radiating pain laterally on the thigh is frequency observed in patients with acetabular retroversion. The most frequent physical finding in retroversion is limited internal rotation during maximal flexion and adduction of the hip. (43, 44). The Drehmann sign (passive external rotation with hip flexion) may also be seen in retroversion, however it may also be present in patients with SCFE or osteoarthritis (45–47). Plain radiographs are the first diagnostic tool used to evaluate acetabular version. The presence of the anterior and posterior acetabular borders crossing (crossover sign) (Figure 3) or deficient posterior acetabular wall observed on anteroposterior hip radiographs are indicative of possible retroversion. However, to measure acetabular version, CT imaging is typically necessary. Using the axial view, acetabular version is the angle formed by a line connecting the anterior and posterior acetabular margins and a perpendicular line that is transverse to the reference line through either the femoral head centers, posterior acetabular walls, or respective posterior aspect of the ischial bones (48). Physiologic acetabular anteversion is approximately 12–20 degrees in adults, however, this can also be variable (40, 49–51).

## Microinstability

Despite the hip being one of the most stable joints in the human body, there is increased recognition of microinstability as a pathologic process associated with significant hip pain (52). Stability of the hip, much like that of any joint, is attributable to its constrained osseous anatomy, acetabular labrum that expands the volume, intracapsular and extracapsular ligamentous structures, and the 17 muscles that traverse the hip joint providing dynamic stability (52). However, pathology to single components may cause instability, without frank dislocations, that contribute to pain within the hip joint.

**Figure 4:**
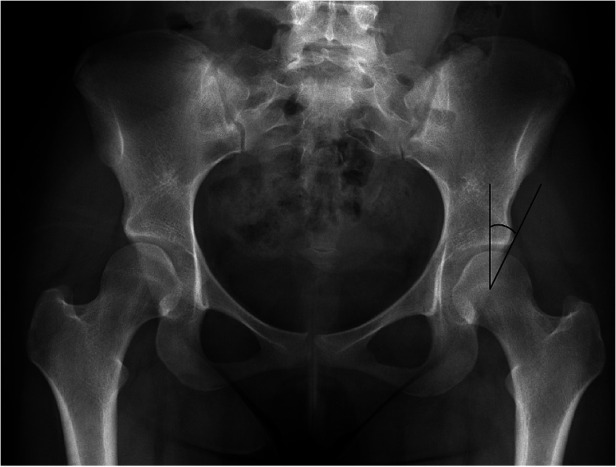
Anteroposterior radiographic image of a pelvis demonstrating left hip dysplasia, which is defined as the lateral center edge angle less than 20^o^.

Osseous abnormalities, most notably in developmental dysplasia of the hip, is a source of likely microinstability of the hip. Patients with mild dysplasia, often termed borderline dysplasia, may often go unnoticed into their adult life, and is reported to be prevalent in 0.1% of the U.S. population (53). The lateral center-edge angle under 20^o^ is often used as a marker for dysplasia (Figure 4). In previous studies, a decreased angle has been noted in 4% of hips with labral tears (54).

There is increased controversy regarding the management of patients with borderline dysplasia. From review of the literature, Kirsch et al. found hip arthroscopy to be a viable option for patients with mild dysplasia, however, outlined contraindications as follows: (1) lateralization of femur >1 cm, break in Shenton's line, lateral center edge angle <20^o^, excess femoral and acetabular anteversion, excess coxa valga, dysplastic proximal femur, tonnis angle >10^o^, and pain with standing and straight-ahead walking (55). Parvizi and colleagues found that patients with dysplasia that underwent arthroscopic surgery had good short term outcomes (at 6 weeks), however, in the long-term, patients had an accelerated rate of osteoarthritis (46.7%), femoral head migration (43.3%), and need for further operative intervention (53.3%) (56).

Ligamentous structures of the hip also provide areas of possible pathology. The ligamentum teres and iliofemoral, pubofemoral, and ischiofemoral ligaments all confer stability to the hip (52). Damage to these ligamentous structures may be increasingly relevant in posttraumatic instability following hip dislocation or acetabular fracture. Alternatively, microinstability of the hip will have a higher differential in patients with history of connective tissue disorders such as Marfan's or Ehrler's Danlos syndromes.

Microinstability of the hip may be a result of FAIS or occur concurrently with the above-mentioned pathologies. Physical examination is a key aspect in identifying patients at risk. Three key provocative test maneuvers are the anterior apprehension test (performed in supination with the examinee holding the contralateral knee flexed to the chest and the examiner passively hyperextending the opposite knee), the abduction-extension-external rotation test (performed in lateral decubitus with the examined leg abducted to 30^o^ and the examiner applying anteriorly directed force to the posterior greater trochanter), and the prone external rotation test (performed prone in neutral hip flexion and the examinee externally rotates the hip with the knee flexed and places anterior directed force on the posterior greater trochanter) (52). If all three tests are positive, there is a 95% likelihood of confirmation intraoperatively of microinstability (57). Magnetic resonance imaging is the key imaging modality for diagnosis of hip instability. However, magnetic resonance arthrograms may also be considered to confirm capsular defects visible with extravasation of fluid (57, 58).

## Conclusion

The treatment of pre-arthritic hip pain is complex and often challenging as it may be multifactorial. While FAIS is a predominant etiology, multiple other considerations should be evaluated prior to surgery. Associated conditions discussed within the present review have the capacity to negatively impact patient outcomes when not addressed. Thereby, it is imperative that thorough physical exam maneuvers and advanced imaging is performed in the preoperative assessment to limit confounding diagnoses in the management of FIAS.
